# Femoral shaft fracture osteosynthesis in a critically ill patient under Extracorporeal Membrane Oxygenation (ECMO)

**DOI:** 10.1051/sicotj/2016012

**Published:** 2016-05-13

**Authors:** Cristobal Calvo, Matias Salineros, Rodrigo Diaz, Sebastian Carvajal

**Affiliations:** 1 Universidad de los Andes School of Medicine 7620001 Santiago Chile; 2 Clinica Las Condes 7591046 Santiago Chile

**Keywords:** Osteosynthesis, Extracorporeal Membrane Oxygenation, ECMO, Orthopedic surgery

## Abstract

*Introduction*: Extracorporeal Membrane Oxygenation (ECMO) is an invasive procedure used in critically ill patients with catastrophic pulmonary failure or cardiogenic shock in which conventional management has failed. These patients are managed with permanent anticoagulation, with increased bleeding risk. Hemorrhage is the main reported complication.

*Case*: A 25-year-old polytraumatized woman, both lower limbs amputated and a left femoral shaft fracture with catastrophic pulmonary failure (Murray score 4) that required intensive management care with ECMO. During her evolution definitive femoral shaft osteosynthesis with a nail as required and the medical team decided to operate on the patient under ECMO. She recovered with fluctuations in her hematocrit, but was hemodynamically stable. The patient recovered satisfactorily, was weaned from ECMO and commenced her rehabilitation program. At 16 months, she was almost autovalent, and full consolidation was achieved, with no complication of the implants.

*Discussion*: ECMO is a life-saving support, but requires permanent anticoagulation, which implies a high risk of hemorrhages, specially for surgical treatment. This patient underwent an osteosynthesis surgery satisfactorily. Hematoma was the only complication of her intramedullary femoral nail, without compromising hemodynamics. This case shows that patients on ECMO can undergo a major orthopedic surgery in selected cases.

## Introduction

Extracorporeal Membrane Oxygenation (ECMO) is an invasive procedure that is used in critically ill patients in whom invasive mechanical ventilation and pharmacological therapies have failed.

Surgery is possible in patients with ECMO, considering the high risk of maintaining anticoagulation therapy during the procedure. Appropriate perioperative management is crucial. Complications of surgery during ECMO include bleeding, hypovolemia, and infections [1].

The challenge in an orthopedic patient under ECMO who requires surgery is related to the increased risk of bleeding, due to the anticoagulation condition [1]. This risk could be reduced with blood-saving techniques. In the case of pelvis, hip, or femur surgery, there are no blood sparing techniques available, making hemorrhages and hypovolemia real surgical risks [2].

## Case

**Figure 1.**
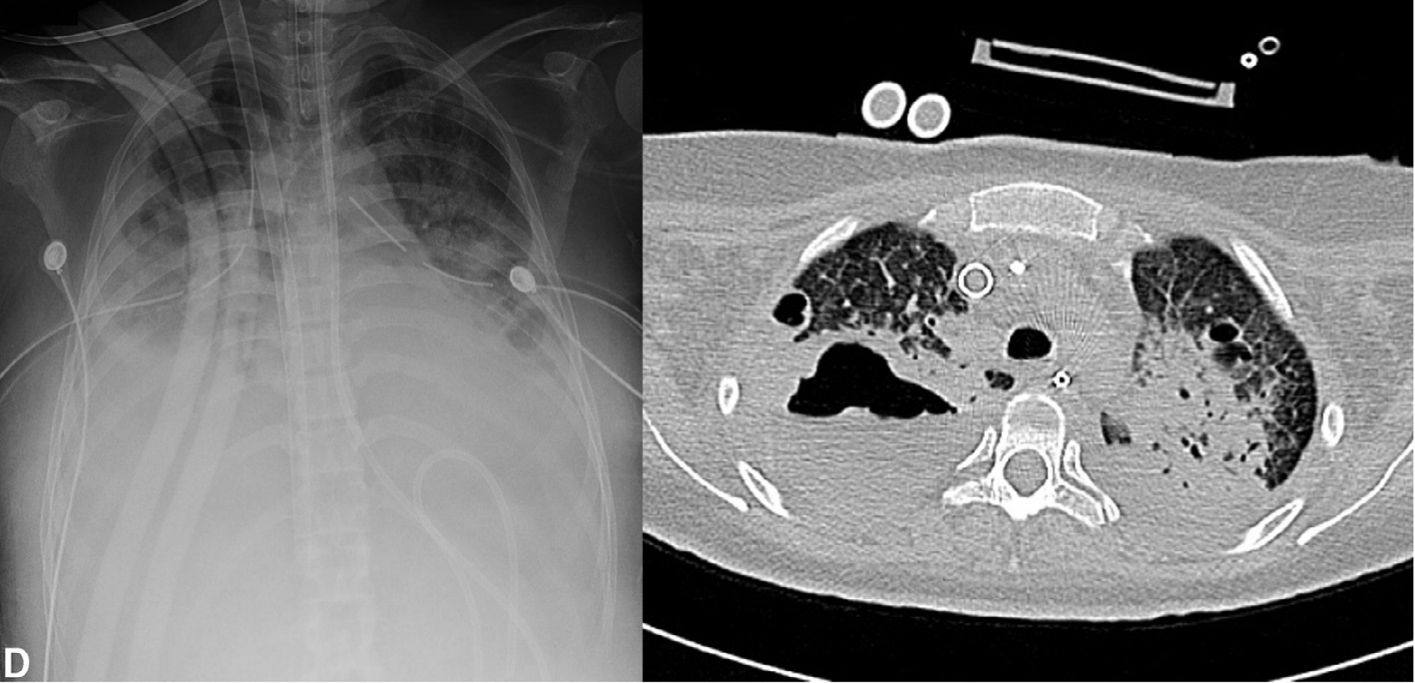
Patient’s lung contusion at the initial evaluation. Thorax anteroposterior radiographs and thoracic CT showing extensive lung contusion.

**Figure 2.**
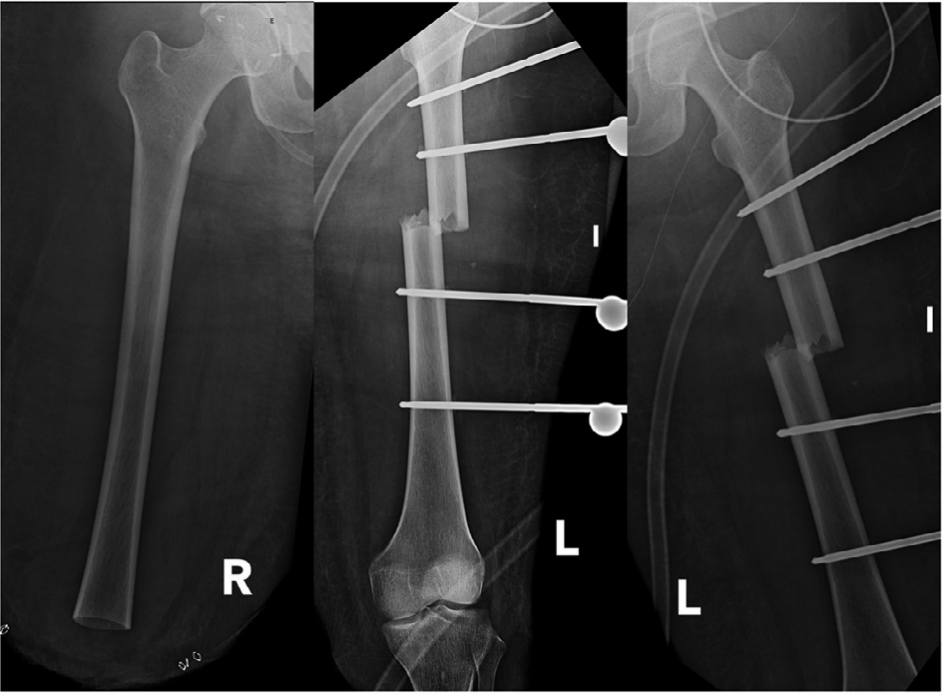
Femoral radiographs after damage control surgery. Right lower limb underwent a supracondylar amputation (left image). Left lower limb underwent a transtibial amputation and a femoral external fixation (middle and right images).

A 25-year-old woman, suffered a car accident, without a safety belt. She was initially managed in a trauma center. The initial examination revealed bilateral lower limbs amputations, left femoral shaft fracture, left pneumothorax of 30% associated with massive pulmonary contusion (Figure 1), scalp wound, C4, C5, left ulna, and right clavicle fractures. She underwent surgery for right supracondylar and left transtibial lower limb amputation. The left femoral shaft fracture was stabilized with an external fixator (Figure 2).

During the postoperative care she developed catastrophic pulmonary failure, with a poor response to prone and high-frequency oscillatory ventilation. Due to this scenario she was transferred to our institution on Veno-Venous ECMO (cannulated in the referral hospital).

She developed an infectious focus on both lower limb stumps (*Pseudomona aeruginosa* and *Proteus mirabilis*). Antibiotic treatment was started with Ceftazidime, Tobramycin, and Linezolid. Hemodynamics remained stable. Stumps were managed with surgical debridement, improving skin coverage using Vacuum-Assisted Closure (VAC) system.

**Figure 3.**
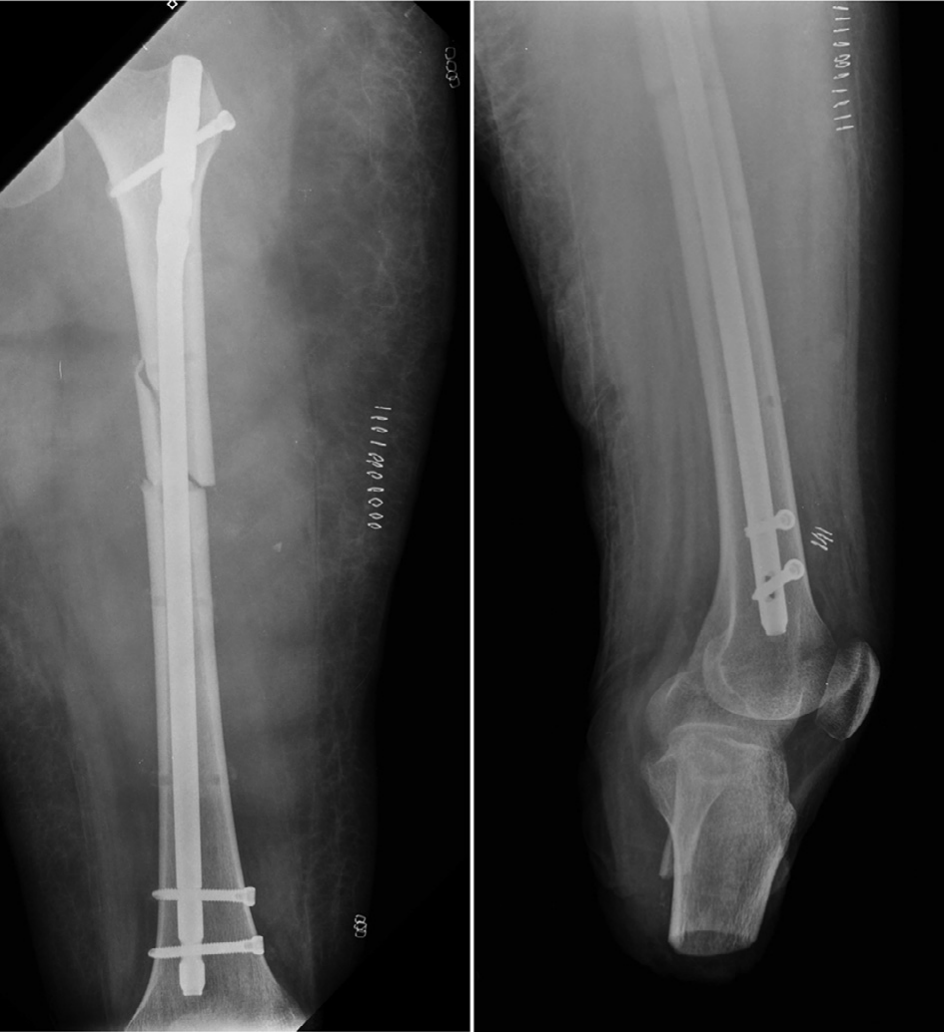
Definitive femoral nail osteosynthesis.

The only definitive treatment for her critical condition to eradicate the cutaneous focus necessitated removing the infected pins. As a multidisciplinary team we decided to perform a definitive osteosynthesis with an antegrade centromedular femoral nail (Smith & Nephew Tan^®^) whilst under ECMO (Figure 3). Anesthesia was based on intravenous drugs as the transfer of volatile anesthetics via ECMO oxygenators is severely limited. Hemodynamic stability was maintained with transfusion (RBC 2 U) and crystalloids. The internal transport of ECMO patients is a routine procedure, as these patients usually need imaging studies or surgical procedures. During surgery the anticoagulation was set to a Partial Thromboplastin Time (PTT) of 45 s, with heparin inflow between 10 and 15 U/kg/h. Tranexamic acid was used during surgery at a 15 mg/kg dose. Two days postoperatively she develops an acute 8% hematocrit fall, receiving two red blood cell units and five cryoprecipitated units. After transfusion hemodynamics was stable without evidence of active bleeding.

The patient recovered well with improving her gasometrics and respiratory condition. The surgical wounds did not develop any complications, and some small hematomas resolved without active management. Surgery was performed on day 15 under ECMO, and the ECMO was used for a total of 22 days. On the seventh day postoperative the ECMO was discontinued and the patient was returned to her hospital to continue rehabilitation. At 16 months, she is almost recovered, and full fracture healing was achieved, with no complication of the implants.

## Discussion

The ECMO is reserved for critically ill patients as a salvage measure. Patients on ECMO are at high surgical risk. Usually, surgical indications are reserved for cardiac procedures. Nevertheless, non-cardiac surgery is not contraindicated in these patients. No reported cases of orthopedic surgery during ECMO are published. An epidural abscess case constitutes the nearest report related to orthopedics [3]. This is the first case reported of an acute trauma patient on ECMO who underwent a major orthopedic surgery, such as a femoral nail.

The benefit of femoral osteosynthesis in a polytraumatized patient is well known [4]. In the septic context of this patient, definitive femoral nail osteosynthesis was the best therapy available. Operative wound hemorrhages are reported as high as 22.4% [5]. In this case, some hematomas occurred but active treatment was not required. The only complication, as expected, was increased perioperative bleeding. Medical care and transfusion therapy were enough for managing this situation.

There are many anesthetic issues to be considered for the surgical procedure [1]. Anticoagulation needed on ECMO is one of the greatest problems for anesthesiologists. Bleeding is a permanent risk, either intraoperatively or postoperatively. In this patient, the goal was to keep a lower anticoagulation threshold and adding antifibrinolytic therapy.

The presented case shows that a polytraumatized critically ill patient on ECMO can undergo major orthopedic surgery successfully, provided that they are well managed in the perioperative period, even if blood sparing techniques are not available.

## Conflict of interest

The authors declare no conflict of interest in relation with this paper.
